# Sex differences in circulating proteins in heart failure with preserved ejection fraction

**DOI:** 10.1186/s13293-020-00322-7

**Published:** 2020-08-24

**Authors:** Susan Stienen, João Pedro Ferreira, Masatake Kobayashi, Gregoire Preud’homme, Daniela Dobre, Jean-Loup Machu, Kevin Duarte, Emmanuel Bresso, Marie-Dominique Devignes, Natalia López Andrés, Nicolas Girerd, Svend Aakhus, Giuseppe Ambrosio, Hans-Peter Brunner-La Rocca, Ricardo Fontes-Carvalho, Alan G. Fraser, Loek van Heerebeek, Gilles de Keulenaer, Paolo Marino, Kenneth McDonald, Alexandre Mebazaa, Zoltàn Papp, Riccardo Raddino, Carsten Tschöpe, Walter J. Paulus, Faiez Zannad, Patrick Rossignol

**Affiliations:** 1 Université de Lorraine, INSERM, Centre d’Investigation Clinique et Plurithématique 1433, INSERM U1116, CHRU de Nancy, F-CRIN INI-CRCT (Cardiovascular and Renal Clinical Trialists), Nancy, France; 2 Department of Physiology and Cardiothoracic Surgery, Cardiovascular Research and Development Unit, Faculty of Medicine, University of Porto, Porto, Portugal; 3 Clinical Research and Investigation Unit, Psychotherapeutic Center of Nancy, Laxou, France; 4 LORIA (CNRS, Inria NGE, Université de Lorraine), Campus Scientifique, F-54506 Vandœuvre-lès-Nancy, France; 5 Navarrabiomed, Complejo Hospitalario de Navarra (CHN), Universidad Pública de Navarra (UPNA), IdiSNA, Pamplona, Spain; 6 Oslo University Hospital, Oslo, Norway; 7 ISB, Norwegian University of Science and Technology, Trondheim, Norway; 8 Division of Cardiology, University of Perugia School of Medicine, Perugia, Italy; 9 Department of Cardiology, Maastricht University Medical Center, Maastricht, the Netherlands; 10 Department of Surgery and Physiology, Cardiovascular Research Unit (UnIC), Faculty of Medicine, University of Porto, Porto, Portugal; 11 Wales Heart Research Institute, Cardiff University, Cardiff, UK; 12 Department of Cardiology, Onze Lieve Vrouwe Gasthuis, Amsterdam, the Netherlands; 13 Laboratory of Physiopharmacology, Antwerp University and ZNA Hartcentrum, Antwerp, Belgium; 14 Clinical Cardiology, Università del Piemonte Orientale, Department of Translational Medicine, Azienda Ospedaliero Universitaria “Maggiore della Carità”, Novara, Italy; 15 St Michael’s Hospital Dun Laoghaire Co. Dublin, Dublin, Ireland; 16 Department of Anaesthesiology and Critical Care Medicine, Saint Louis and Lariboisière University Hospitals and INSERM UMR-S 942, Paris, France; 17 Division of Clinical Physiology, Department of Cardiology, Faculty of Medicine, University of Debrecen, Debrecen, Hungary; 18 Department of Cardiology, Spedali Civili di Brescia, Brescia, Italy; 19 Department of Cardiology, Campus Virchow-Klinikum, Charite Universitaetsmedizin Berlin, Berlin Institute of Health – Center for Regenerative Therapies (BIH-BCRT), and the German Center for Cardiovascular Research (DZHK ; Berlin partner site), Berlin, Germany; 20 Amsterdam Cardiovascular Sciences, Amsterdam University Medical Centers, Amsterdam, the Netherlands

## Abstract

**Background:**

Many patients with heart failure with preserved ejection fraction (HFpEF) are women. Exploring mechanisms underlying the sex differences may improve our understanding of the pathophysiology of HFpEF. Studies focusing on sex differences in circulating proteins in HFpEF patients are scarce.

**Methods:**

A total of 415 proteins were analyzed in 392 HFpEF patients included in The Metabolic Road to Diastolic Heart Failure: Diastolic Heart Failure study (MEDIA-DHF). Sex differences in these proteins were assessed using adjusted logistic regression analyses. The associations between candidate proteins and cardiovascular (CV) death or CV hospitalization (with sex interaction) were assessed using Cox regression models.

**Results:**

We found 9 proteins to be differentially expressed between female and male patients. Women expressed more LPL and PLIN1, which are markers of lipid metabolism; more LHB, IGFBP3, and IL1RL2 as markers of transcriptional regulation; and more Ep-CAM as marker of hemostasis. Women expressed less MMP-3, which is a marker associated with extracellular matrix organization; less NRP1, which is associated with developmental processes; and less ACE2, which is related to metabolism. Sex was not associated with the study outcomes (adj. HR 1.48, 95% CI 0.83–2.63), *p* = 0.18.

**Conclusion:**

In chronic HFpEF, assessing sex differences in a wide range of circulating proteins led to the identification of 9 proteins that were differentially expressed between female and male patients. These findings may help further investigations into potential pathophysiological processes contributing to HFpEF.

## Introduction

Heart failure with preserved ejection fraction (HFpEF) presents an important challenge for clinical practice given its rising incidence, poor prognosis, and lack of clear evidence from randomized clinical trials of treatment that can reduce mortality [1–4]. Consistent epidemiological data demonstrate that women constitute the majority of patients with HFpEF [5]. Although to some extent explained by sex differences in cardiovascular structure and function, immune system biology, and the myocardial response to comorbidities [6], the underlying pathophysiological mechanisms of HFpEF remain incompletely understood.

Given the sex differences in HFpEF, we sought differences in biomarker profiles possibly linked to underlying pathophysiology. In this study, we therefore assessed sex differences in 415 circulating proteins in a prospective observational cohort of HFpEF patients (The Metabolic Road to Diastolic Heart Failure (MEDIA-DHF) study; NCT02446327).

## Methods

### Study population

In MEDIA-DHF, a multicenter, multinational, observational study, a total of 626 HFpEF patients were enrolled between 2012 and 2014 in 10 centers (listed in Supplemental table 1). A description of this study population and methods has been published previously [7]. To summarize, after standardized echocardiography (Supplemental data) and/or local natriuretic peptide measurements, eligible patients with a diagnosis of diastolic dysfunction as established by the 2007 ESC recommendations [8] were included. Three clinical modes of presentations were considered: (i) acute decompensated HF patients, (ii) patients recently discharged after admission for an acute HF episode (< 60 days), or (iii) ambulatory chronic disease patients. Data on demographics, clinical parameters, laboratory values (including extensive biomarker measurements), electrocardiography, and echocardiography were obtained at inclusion in the study. Follow-up visits took place at 3, 6, and 12 months after inclusion in the study.

Only patients in whom the circulating proteins were measured were included in this substudy (*N* = 392; see also flowchart in Supplemental figure 1). No protein measurements were performed in acute decompensated HF patients.

### Biomarker measurements

Plasma samples taken at inclusion in the study were analyzed for circulating protein (including natriuretic peptides) using the Olink Proseek Multiplex cardiovascular disease (CVD) II, III, inflammation, cardiometabolic, and organ damage panels (Olink Proteomics, Uppsala, Sweden). The assay uses a proximity extension assay (PEA) technology where 92 oligonucleotide-labeled antibody probe pairs per panel are allowed to bind to their respective targets in 1 μL plasma sample. The PEA technology has been described previously [9]. In brief, when binding to their correct targets, they give rise to new DNA amplicons with each ID barcoding their respective antigens. The amplicons are subsequently quantified using a Fluidigm BioMark HD real-time PCR platform. Data is quality controlled and normalized using an internal extension control and an inter-plate control, to adjust for intra- and inter-run variation. The extension control is composed of an antibody coupled to a unique pair of DNA-tags that serves as a synthetic control that is added to every sample well. It will adjust for technical variation introduced in the extension step and hence reduce intra-assay variability. The final assay read-out is presented in log2-normalized protein expression (NPX) data where an increase of 1 NPX confers a doubling in concentration of the specific biomarker. This arbitrary unit is therefore a relative quantification of proteins and compares fold changes between groups. All assay validation data for the proteins in the cardiovascular II, cardiovascular III, inflammation, cardiometabolic, and organ damage panels (detection limits, intra- and inter-assay precision data, accuracy, etc.) are available on the manufacturer’s website (www. olink.com). We excluded proteins that were below the lower limit of detection in more than 50% of the patients (*N* = 45). For the proteins below the LOD in less than 50% of patients, the LOD value was imputed. A total of 415 protein circulating proteins were studied. The abbreviations, full names, and respective Olink multiplex panels of all measured proteins are described in the Supplemental table 2.

### Clinical outcome

The pre-specified endpoint of MEDIA-DHF was a composite of CV death and/or CV hospitalizations (NCT02446327). All endpoints were adjudicated by an independent endpoint committee blinded to the biomarker data.

### Network analyses

The FHF-GKB (Fight Heart Failure-Graph Knowledge Box) resource is a customized upgrade of the EdgeBox provided by the EdgeLeap company (available from: https://www.edgeleap.com/edgebox). It extracts data from public data sources hence providing most available public knowledge about human protein-disease, protein-protein, and protein-pathway relationships. The FHF-GKB resource enables the study of a total of 20,386 protein nodes imported from UniProt [10] (including all circulating proteins involved in this study), 28,176 disease nodes from Disease Ontology [11] and DisGenet [12], and 2222 pathway nodes from Reactome (v65) [13]. Protein–protein relationships were retrieved from STRING (v10.5) [14], or Reactome or WikiPathways [15], or Mentha [16], or BioGrid [17], protein–disease associations from DisGenet (2018-08-24), and protein–pathway relationships from Reactome.

### Statistical analyses

Baseline clinical, demographic, and echocardiographic characteristics were compared between male and female patients using chi-square, *t* test, or Mann-Whitney tests, as appropriate.

To study whether biomarker patterns differ between male and female patients, we first identified which clinical variables were associated with female sex using logistic regression analysis (“clinical model”). All variables listed in Table 1 were considered in univariate analyses, apart from dyslipidemia as a diagnosis, hemoglobin as a continuous variable, NT-proBNP, and use of aspirin. Variables with ≥ 20% missing values were excluded from further analyses (pulmonary artery pressure (PASP), left atrial area, ratio of the early (E) to late (A) ventricular filling velocities (E/A), waist circumference, sodium, potassium, and CRP). Anemia was defined as hemoglobin < 12 g/dL in women and < 13 g/dL in men, according to the World Health Organization recommendations.

**Table 1:** Baseline characteristics of patients in the MEDIA-DHF cohort according to sex

	Global (***N*** = 392)	Male (***N*** = 142)	Female (***N*** = 250)	***p*** value	% of missing values
Age, years, median (IQR)	74.0 (67.5–80.0)	73.0 (67.0–79.0)	75.0 (68.0–81.0)	0.028	0
Recently decompensated HF, *n* (%)	60 (15.3%)	25 (17.6%)	35 (14.0%)	0.34	0
Smoking status, *n* (%)					
Never	205 (52.7%)	39 (27.9%)	166 (66.7%)	< 0.001	1
Former	150 (38.6%)	84 (60.0%)	66 (26.5%)		
Current	34 (8.7%)	17 (12.1%)	17 (6.8%)		
BMI, kg/m^2^, mean ± SD	30.6 ± 6.2	30.5 ± 5.6	30.6 ± 6.5	0.79	1
Alcohol status, *n* (%)					
Non-consumer	245 (63.3%)	73 (52.1%)	172 (69.6%)	0.001	1
1–2 drinks/day	124 (32.0%)	56 (40.0%)	68 (27.5%)		
>2 drinks/day	18 (4.7%)	11 (7.9%)	7 (2.8%)		
Waist circumference, cm, mean ± SD	104.8 ± 14.1	108.2 ± 13.8	102.9 ± 13.9	0.001	21
SBP, mmHg, mean ± SD	137.2 ± 23.1	137.5 ± 22.3	137.0 ± 23.5	0.83	2
DBP, mmHg, mean ± SD	74.0 ± 11.6	74.7 ± 11.1	73.7 ± 11.9	0.41	2
Pulmonary rales, *n* (%)	89 (23.0%)	33 (23.4%)	56 (22.8%)	0.89	1
NYHA class III/IV, *n* (%)	73 (18.7%)	23 (16.2%)	50 (20.1%)	0.34	0
Peripheral edema, *n* (%)	178 (45.4%)	74 (52.1%)	104 (41.6%)	0.045	1
Elevated JVP, *n* (%)	24 (6.3%)	12 (8.7%)	12 (4.9%)	0.15	3
Hepatomegaly, *n* (%)	13 (3.6%)	6 (4.5%)	7 (3.1%)	0.68	7
Fatigue at exertion, *n* (%)	305 (79.2%)	105 (75.5%)	200 (81.3%)	0.18	2
Heart rate, bpm, mean ± SD	69.4 ± 14.4	67.1 ± 12.9	70.8 ± 15.0	0.016	3
Hypertension, *n* (%)	342 (87.7%)	121 (86.4%)	221 (88.4%)	0.57	1
Atrial fibrillation, *n* (%)	117 (31%)	49 (36%)	68 (28%)	0.13	3
Diabetes mellitus, *n* (%)	154 (39.3%)	67 (47.2%)	87 (34.8%)	0.016	0
Dyslipidemia, *n* (%)	226 (58.2%)	75 (53.6%)	151 (60.9%)	0.16	1
Previous HF hospitalization, *n* (%)	137 (35.5%)	65 (46.8%)	72 (29.1%)	< 0.001	2
CAD, *n* (%)	128 (33.7%)	73 (52.9%)	55 (22.7%)	< 0.001	3
Stroke or TIA, *n* (%)	44 (11.3%)	14 (10.0%)	30 (12.0%)	0.54	1
Peripheral artery disease, *n* (%)	34 (8.9%)	18 (13.0%)	16 (6.5%)	0.030	2
COPD, *n* (%)	71 (18.4%)	30 (21.4%)	41 (16.7%)	0.25	2
Laboratory values, mean ± SD					
LDL, mg/dL	99.8 ± 37.1	89.8 ± 30.2	104.5 ± 39.1	< 0.001	20
HDL, mg/dL	53.7 ± 19.7	47.3 ± 15.5	56.8 ± 20.8	< 0.001	19
Total cholesterol, mg/dL	176.1 ± 43.1	160.2 ± 35.1	183.9 ± 44.6	< 0.001	17
Hemoglobin, g/dL	13.0 ± 1.6	13.4 ± 1.8	12.8 ± 1.5	< 0.001	12
Anemia, *n* (%)	106 (30.6%)	46 (39.0%)	60 (26.3%)	0.019	12
eGFR, mL/min/1.73 m^2^	66.4 ± 23.1	66.4 ± 23.7	66.4 ± 22.8	1.00	6
NT-proBNP (in NPX)	4.2 ± 1.3	4.4 ± 1.4	4.1 ± 1.3	0.042	0
Medication prescription rates, *n* (%)					
ACEi or ARB	319 (81.4%)	114 (80.3%)	205 (82.0%)	0.67	0
Beta blockers	286 (73.0%)	111 (78.2%)	175 (70.0%)	0.080	0
Thiazide diuretics	87 (22.3%)	22 (15.5%)	65 (26.1%)	0.015	0
Loop diuretics	235 (59.9%)	89 (62.7%)	146 (58.4%)	0.41	0
MRA	43 (11.0%)	16 (11.3%)	27 (10.8%)	0.87	0
Aspirin	159 (40.6%)	64 (45.1%)	95 (38.0%)	0.17	0
Insulin	52 (13.3%)	26 (18.3%)	26 (10.4%)	0.028	0
Statin	241 (61.5%)	96 (67.6%)	145 (58.0%)	0.060	0
Oral anticoagulants	161 (41.1%)	57 (40.1%)	104 (41.6%)	0.78	0

*Legend*: *HF* heart failure, *BMI* body mass index, *DM* diabetes mellitus, *CAD* coronary artery disease, *PAD* peripheral artery disease, *COPD* chronic obstructive pulmonary disease, *OSAS* obstructive sleep apnea syndrome, *TIA* transient ischemic attack, *DBP* diastolic blood pressure, *SBP* systolic blood pressure, *NYHA* New York Heart Association, *JVP* jugular venous pressure, *eGFR* estimated glomerular filtration rate, *BNP* brain natriuretic peptide, *NT-proBNP N*-terminal pro-brain natriuretic peptide, *NPX* normalized protein expression, *ASA* acetylsalicylic acid, *ACEi* ACE-inhibitor, *ARB* angiotensin receptor blocker, *BB* beta blocker, *MRA* mineralocorticoid receptor antagonist, *LVEF* left ventricular ejection fraction, *E/e’* the ratio of mitral inflow velocity and early mitral annulus velocity, *PASP* pulmonary artery systolic pressure, *TAPSE* tricuspidal annular plane systolic excursion, *E/A* ratio of the early (E) to late (A) ventricular filling velocities, *LVEDVi* left ventricular end-diastolic volume index, *LVESVi* left ventricular end-systolic volume index, *LAVI* left atrial volume index, *IQR* interquartile range, *SD* standard deviation

Missing predictor values with < 20% of missing values were imputed using linear regression analyses (see Table 1 for the percentage of missing data for each variable). We imputed missing data 10 times, performed the analysis over all the 10 imputations, and averaged results using Rubin’s rules [18]. Log-linearity of continuous variables was assessed visually by inspecting the shape of the distribution of the beta-estimates vs. the median by quintiles with regard to the outcome of interest (“female sex”). If deemed appropriate based on log-linearity, continuous variables were categorized. Variables with significant *p* values (< 0.05) in univariate analyses were considered in the multivariate model. The discrimination of the final multivariate model for estimation of clinical end-points was assessed by calculating the area under the curve (AUC). Similar beta-estimates of variables in the multivariate model derived from the multiple imputation datasets were obtained when the pooled mean hemoglobin (*N* = 46), heart rate (*N* = 12), and total cholesterol levels (*N* = 67) were imputed in the original datasets (data not shown). Further analyses were therefore performed in the original dataset with pooled mean levels for hemoglobin, heart rate, and total cholesterol.

To identify differences in proteins between sexes, we studied which proteins were significantly associated with female sex on top of the clinical model. Therefore, all proteins were tested individually on top of the clinical model (i.e., fully adjusted on the clinical variables) in a bivariate logistic regression model, correcting for multiple comparisons using a Bonferroni adjusted *p* value of < 0.00012 (alpha divided by 415 proteins). We subsequently searched for correlated proteins (> 0.3). In order to ascertain independence, we excluded the protein with the largest mean absolute correlation from the two correlated proteins. An additional analysis excluding proteins correlated > 0.5 was performed.

The FHF-GKB complex network was queried in order to explore pathways and proteins that could connect together biomarker (BM) nodes of interest. Queries were expressed according to query patterns defining a path structure between two nodes such as BM-BM, BM-pathway-BM, and BM-protein-BM, where the BM nodes are taken from a list of interest. The resulting graphs were merged in a figure illustrating all possible paths not longer than two edges, connecting proteins through pathways and proteins.

The association between sex and the composite endpoint of CV death or CV hospitalizations within 1 year after inclusion in the study was studied using Kaplan–Meier estimates and Cox proportional hazards models. In multivariate analyses, adjustment was performed for clinical variables previously found to be independently associated with CV death or CV hospitalizations in MEDIA-DHF [7]. These clinical variables were a history of CAD, pulmonary rales at baseline, and age. The proportional hazard assumption was not met, and a landmark analysis was performed excluding those patients with an endpoint or being censored within 120 days after inclusion in the study.

Statistical analyses were performed using SPSS 24 (IBM Inc., Armonk, NY) and R (The R Foundation for Statistical Computing, Vienna, Austria). This is the first report on sex differences in clinical features, circulating proteins and outcome in MEDIA-DHF.

## Results

### Baseline characteristics

There were more female than male patients included in MEDIA-DHF (64% vs. 36%, *p* < 0.001). The distribution of demographic, clinical, and laboratory variables according to sex is summarized in Table 1. Female patients were older by an average of 2 years, had lower hemoglobin levels (by 4.5%), and a higher heart rate (by 3 bpm) and total cholesterol (by 120 mg/dL) when compared to male patients. Male patients were more often current smokers and more likely to have a history of diabetes mellitus, CAD, peripheral arterial disease, and prior hospitalizations for HF. Echocardiographic variables are listed in Table 2.

**Table 2:** Echocardiography characteristics of patients in the MEDIA-DHF cohort according to sex

Echocardiographic variables, mean ± SD	Global (***N*** = 392)	Male (***N*** = 142)	Female (***N*** = 250)	***p*** value	% of missing values
LVEF, %	60.8 ± 7.0	60.5 ± 6.9	61.0 ± 7.0	0.54	0
E/e’	13.3 ± 5.2	12.6 ± 4.6	13.7 ± 5.4	0.057	7
PASP, mmHg	34.8 ± 12.7	34.9 ± 15.0	34.8 ± 11.4	0.97	25
TAPSE, cm	20.5 ± 4.8	21.0 ± 5.4	20.2 ± 4.4	0.13	10
E/A	1.3 ± 0.9	1.4 ± 1.0	1.2 ± 0.8	0.033	30
LDEVi	43.8 ± 14.0	48.3 ± 14.4	41.1 ± 13.1	< 0.001	4
LDESi	17.4 ± 7.3	19.6 ± 7.5	16.2 ± 6.7	< 0.001	6
LAVI, ml/m^2^	43.8 ± 15.8	45.0 ± 16.6	43.1 ± 15.4	0.27	3

*Legend*: *LVEF* left ventricular ejection fraction, *E/e’* the ratio of mitral inflow velocity and early mitral annulus velocity, *PASP* pulmonary artery systolic pressure, *TAPSE* tricuspidal annular plane systolic excursion, *E/A* ratio of the early (E) to late (A) ventricular filling velocities, *LVEDVi* left ventricular end-diastolic volume index, *LVESVi* left ventricular end-systolic volume index, *LAVI* left atrial volume index, *IQR* interquartile range, *SD* standard deviation

### Clinical model: variables associated with female sex

Baseline clinical variables that were independently associated with female sex are depicted in Table 3. These variables were higher heart rate, higher total cholesterol, lower hemoglobin, no CAD, no prior HF hospitalizations, and no history of smoking.

**Table 3:** Adjusted and multiple testing-corrected circulating proteins associated with female sex

	Clinical model (AUC 0.82)		Proteins	
	OR (95% CI) for female sex association	*p* value	OR (95% CI) for female sex association	*p* value
Heart rate, per 10 bpm increase	1.22 (1.00–1.48)	0.049		
Total cholesterol, per 10 mg/dL increase	1.13 (1.03–1.02)	0.010		
Smoking status				
Never	Ref.	–		
Former	0.21 (0.12–0.36)	< 0.001		
Current	0.33 (0.14–0.80)	0.014		
CAD	0.36 (0.21–0.62)	< 0.001		
Previous HF hospitalization	0.47 (0.27–0.82)	0.008		
Hb, per 1 g/dL increase	0.69 (0.57–0.82)	< 0.001		
Higher expression in females vs. males				
IL1RL2			4.13 (2.22–7.68)	< 0.001
LPL			4.08 (2.44–6.82)	< 0.001
LHB			3.15 (2.20–4.52)	< 0.001
IGFBP3			2.83 (1.74–4.58)	< 0.001
PLIN1			2.44 (1.62–3.66)	< 0.001
Ep-CAM			1.87 (1.41–2.48)	< 0.001
Less expression in females vs. males				
NRP1			0.05 (0.01–0.19)	< 0.001
MMP-3			0.22 (0.14–0.35)	< 0.001
ACE2			0.41 (0.28–0.61)	< 0.001

Each OR unit increase represents doubling in the NPX values

*Legend*: *eGFR* estimated glomerular filtration rate, *OR* odds ratio, *CI* confidence interval, *AUC* area under the curve, *bpm* beats per minute, *CAD* coronary artery disease, *HF* heart failure, *Hb* hemoglobin, *IGFBP3* insulin-like growth factor-binding protein 3, *NRP1* neuropilin 1, *IL1RL2* interleukin-1 receptor-like 2, *LPL* lipoprotein lipase, *ACE2* angiotensin-converting enzyme 2, *Ep_CAM* epithelial cell adhesion molecule, *CA14* carbonic anhydrase 14, *PLIN1* perilipin-1, *LHB* lutropin subunit beta

### Association between circulating proteins and female sex

For all individual proteins (including NT-proBNP), associations with female sex were assessed. A total of 17 were identified of which 9 proteins were not correlated (*r* < 0.3) with other proteins. Of these 9 proteins, 6 were positively associated with female sex (insulin-like growth factor-binding protein 3 (IGFBP3), interleukin-1 receptor-like 2 (IL1RL2), lipoprotein lipase (LPL), epithelial cell adhesion molecule (Ep-CAM), perilipin-1 (PLIN1), and lutropin subunit beta (LHB)) and 3 were negatively associated (neuropilin-1 (NRP1), matrix metalloproteinase-3 (MMP-3), angiotensin-converting enzyme 2 (ACE2)) with female sex (Table 3; Supplemental Table 2). Proteins with moderate correlations (*r* < 0.5) are shown in Supplemental Table 3.

### Network analyses

**Fig. 1:**
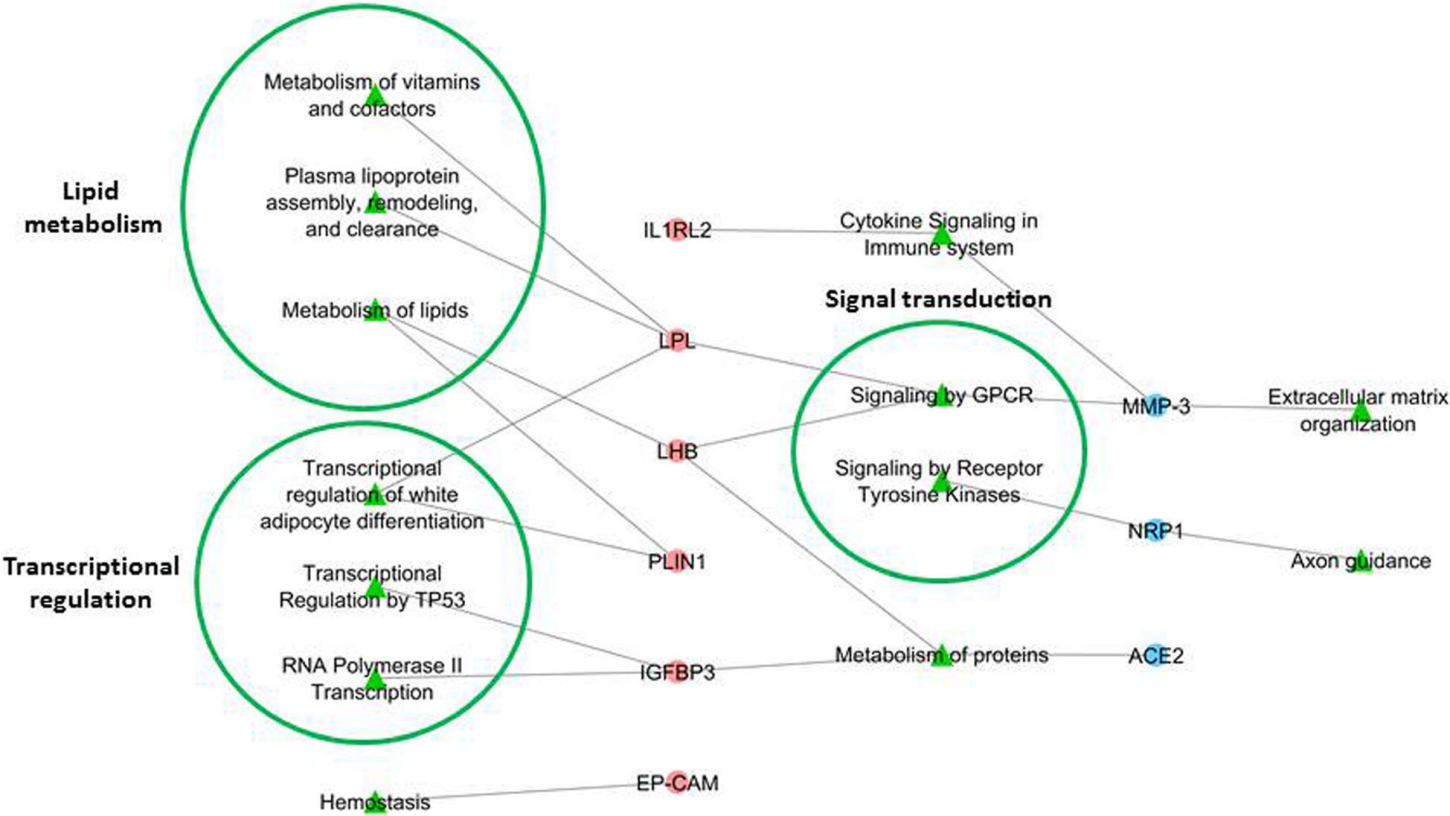
**Network analyses for the visualization of pathways and protein interactions between circulating proteins associated with sex in MEDIA-DHF.** The proteins that were higher expressed in female vs. male patients may be linked to pathways involved in lipid metabolism, transcriptional regulation, and hemostasis. The proteins that were higher expressed in male vs. female patients may be linked to pathways such as extracellular matrix organization and developmental processes. Common pathways between the proteins seem signal transduction, protein metabolism and cytokine signaling. The FHF-GKB complex network was queried in order to explore pathways and proteins that could connect together biomarker nodes of interest. Queries were expressed according to query patterns defining a path structure between two nodes such as BM-BM, BM-pathway-BM, and BM-pathway, where the BM nodes are taken from a list of interest. The resulting graphs were merged in a figure illustrating all possible paths not longer than two edges, connecting proteins through pathways and proteins. This graph depicts biomarker-pathway—protein interactions. There were no direct interactions between proteins. Blue: proteins higher expressed in males. Pink: proteins higher expressed in females. Green: pathways

The visualization of the interactions between circulating proteins and pathways is depicted in the Fig. 1. The 6 proteins that were higher expressed in female vs. male patients were linked to pathways involved in lipid metabolism, transcriptional regulation, and hemostasis. The proteins that were higher expressed in male vs. female patients pointed to pathways related to extracellular matrix organization and developmental processes. Common pathways between the “female” and “male” proteins were signal transduction cascades, protein metabolism, and cytokine signaling.

### Outcome

**Fig. 2:**
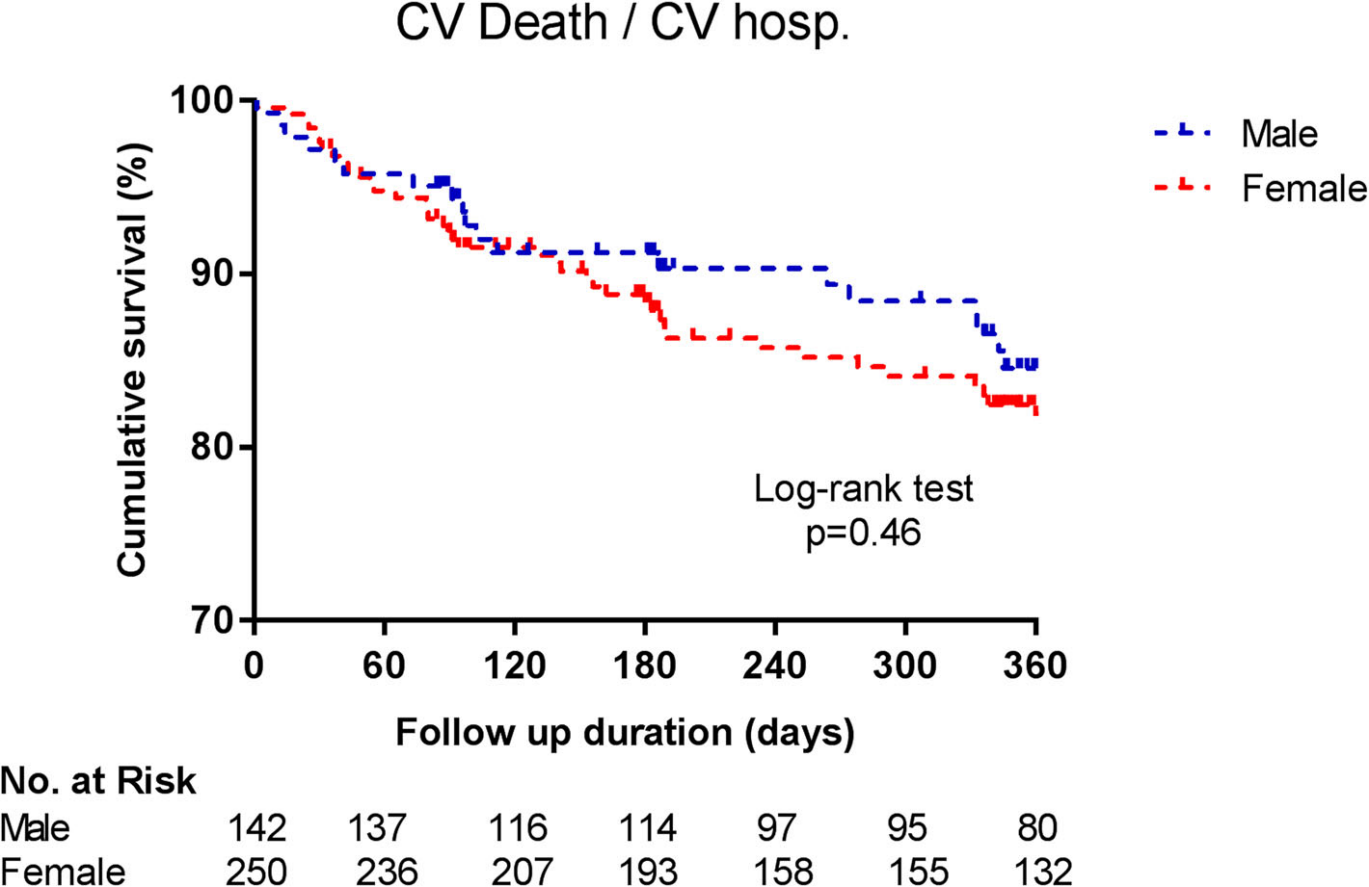
**Primary outcome according to sex.** Legend: CV, cardiovascular; No., number

There was no difference in the rates of the composite endpoint of CV death or CV hospitalization between female and male patients after 1 year of follow-up (event rates 41/250 (16.4%) female patients vs. 19/142 (13.4%) male patients, log rank test: *p* = 0.46; Fig. 2). Crude and adjusted hazard ratios for the composite endpoint are depicted in Table 4 (adjusted HR 1.48, 95% CI 0.83–2.63, *p* = 0.18).

**Table 4:** Crude and adjusted hazard ratios for the prediction of cardiovascular death and/or cardiovascular hospitalization during 1 year of follow-up according to sex

	Crude HR (95% CI)	Adj. HR (95% CI)*
Complete cohort		
Female vs. male	1.26 (0.73–2.16), *p* = 0.41	1.48 (0.83–2.63), *p* = 0.18
Landmark analysis (> 120 days)		
Female vs. male	1.70 (0.72–4.03), *p* = 0.23	1.89 (0.77–4.65), *p* = 0.17

The proportional hazard assumption was not met, and a landmark analysis was performed excluding those patients with an endpoint or being censored within 120 days after inclusion in the study

*Adjusted on coronary artery disease, pulmonary rales at baseline, and age. Since our aim was to assess the association of female sex on top of this clinical risk score and female sex was already incorporated in the prognostic risk score, sex was not considered in the prognostic risk score used for these analyses

## Discussion

In this study, we investigated sex differences in circulating proteins measured in a cohort of 392 HFpEF patients. From over 400 proteins measured, we found 9 proteins to be significantly and independently associated with female sex and HFpEF. Females expressed more IGFBP3, IL1RL2, LPL, Ep-CAM, PLIN1, and LHB which are markers of lipid metabolism, transcriptional regulation, and hemostasis, whereas they expressed less NRP1, MMP-3, and ACE2 which are markers associated with extracellular matrix organization and developmental processes.

### Sex differences: clinical features

We found several clinical variables associated with female subjects with HFpEF: higher heart rate, higher total cholesterol, lower hemoglobin, absence of CAD, no prior HF hospitalizations, and not smoking. These findings are in agreement with previous studies [6, 19, 20]. Although previously shown for acute HF patients [21], an association between female sex and higher cholesterol levels in HFpEF has thus far not been reported. Although females were slightly older than male subjects, there was no significant association between age and female sex in this cohort. Interestingly, females were 14% less likely to receive statin therapy at baseline in MEDIA-DHF compared to males. A possible explanation for the higher total cholesterol levels but lower rates of CAD in females compared to males may be that females tend to have microvascular lesions and endothelial dysfunction, whereas males are more likely to have obstructive coronary lesions [22], which may potentially lead to an underdiagnosis of coronary artery disease in females.

### Sex differences: circulating proteins

In this study, plasma levels of 9 proteins were significantly differentially expressed between female and male patients. Of these 9 proteins, 6 were higher expressed in female compared to male patients (IGFBP3, IL1RL2, LPL, Ep-CAM, PLIN1, and LHB), whereas the other 3 proteins were higher expressed in males (NRP1, ACE2, and MMP-3). Network analysis revealed that the proteins that were higher expressed in female vs. male patients may be linked to pathways involved in lipid metabolism, transcriptional regulation, and hemostasis. The proteins that were higher expressed in males vs. females pointed more towards pathways involved in extracellular matrix organization and developmental processes. Common pathways were signal transduction cascades, protein metabolism, and cytokine signaling.

Proposed mechanistic actions and previous studies investigating the differently expressed circulating proteins in HFpEF are described next and summarized in Table 5. IGFBP3 is the most abundant carrier protein for insulin-like growth factor 1 (IGF-1) which is known to regulate proliferation, differentiation, metabolism, and cell survival in various tissues. It has been linked to hypertension, obesity, cardiovascular disease, and many cancers [27, 28]. After an acute myocardial infarction, IGFBP-3 and IGF-1 levels significantly increased, which was associated with improved outcomes and echocardiographic parameters (LV dimensions, mass and ejection fraction) [30]. A recent study in 84 HFpEF patients demonstrated a high prevalence of anabolic hormonal deficiencies (including IGF-1), and low IGF-1 serum levels were associated with increased left atrial size and volume [29]. Although IGFBP-3 levels were numerically higher in female compared to male participants in the Cardiovascular Health Study [31], to our knowledge, there have been no reports, so far, on sex differences in HFpEF or other cardiovascular diseases.

**Table 5:** Overview of the proteins differentially expressed between sexes

Individual biomarker	Mechanistic significance	Previous relevant reports		Reports on sex differences	
		Basic reports	Clinical reports	Healthy population	Heart disease
More highly expressed in females					
IL1RL2	Activates pro-inflammatory pathways upon binding of IL-36 [23]		Involved in inflammatory diseases such as psoriasis, inflammatory bowel disease and rheumatoid arthritis [23]	No	No
LPL	Enzyme that catalyzes the hydrolysis of triglycerides	Overexpression or downregulation of cardiac LPL in diabetic mouse models resulted in impaired left ventricular function [24].	Associated with coronary heart disease, Alzheimer disease, and chronic lymphocytic leukemia [25]	No	No
LHB	Promotes spermatogenesis and ovulation by stimulating the testes and ovaries to synthesize steroids		Levels not different between CAD and control subjects [26]	Yes (higher in females vs. males [26])	Yes (higher in female vs. male CAD patients [26])
IGFBP3	Most abundant carrier protein for insulin-like growth factor 1 (IGF-1) which is known to play a major role in metabolism	The IGF system (including IGFBP3) has been previously associated with cardiovascular disease and many cancers [27, 28]	In HFpEF patients, low IGF-1 and IGFBP3 were associated with increased parameters of left atrial size and volume [29]. Higher IGF-1 after an acute MI was associated with improved clinical outcomes and echocardiographic measures (LV dimensions, mass, and ejection fraction) [30]	Yes (numerically higher in female vs. male but no formal comparison made [31])	No
PLIN1	Surface protein of adipocyte lipid droplets that regulates storage and hydrolysis of adipose triglycerides [32]	Linked to endocrine metabolism disease (diabetes, obesity etc.), cancers, and cardiovascular disease [33]	Higher expressed in the right atria of patients with CAD compared to those without [34]	No	No
Ep-CAM	Transmembrane glycoprotein expressed in epithelium	Involved in various processes such as cell signaling, cell-cell adhesion, proliferation and differentiation, tumorigenesis, and metastasis of carcinomas [35]	Not associated with adverse cardiovascular outcomes in a registry of 263 chronic heart failure patients [36] Associated with inflammatory bowel disease [35]	No	No
Less expressed in females					
NRP1	Transmembrane receptor for class III semaphorins and for members of the vascular endothelial growth factor family [37]	Neuronal and vascular development during embryogenesis, angiogenesis, and maintenance of vascular integrity [38]	Associated with poor outcome in HFpEF but not in HFrEF patients [39]	No	No
MMP-3	Enzyme involved in the breakdown of extracellular matrix proteins	Involved in physiological (e.g. embryogenesis) and pathophysiological (e.g. tumor metastasis and atherosclerosis) processes [40]	Conflicting data: high levels have been described in atherosclerotic plaques [41] and associated with poor outcome after MI [42]. However, a common mutation in the MMP-3 promoter (which results in decreased MMP-3 expression) was associated with atherosclerosis development [43]	No	Yes (higher in male vs. female patients post-MI [42])
ACE2	Hydrolyses angiotensin I and angiotensin II generating angiotensin (1-9) and angiotensin [1–5], respectively.		ACE2 levels were higher in patients with type 1 diabetes and coronary heart disease vs. controls [44]	Yes (conflicting data: similar levels in male and females [45] or higher in male vs. female [44])	Yes (higher in males vs. females with type 1 DM and CAD [44])

IL1RL2 (or IL-36 receptor) activates pro-inflammatory pathways upon binding to IL-36 [23]. Accumulating data indicates that IL1RL2 is involved in inflammatory diseases such as psoriasis, inflammatory bowel disease, and rheumatoid arthritis [23]. No previous studies have reported on IL1RL2 in HFpEF or other cardiovascular disease.

The transmembrane glycoprotein expressed in epithelium, Ep-CAM, is involved in various processes such as cell signaling, cell–cell adhesion, proliferation and differentiation, tumorigenesis, and metastasis of carcinomas and has been associated with inflammatory bowel disease [35]. Ep-CAM was not associated with adverse cardiovascular outcomes in a registry of 263 chronic heart failure patients [36]. To our knowledge, no studies have investigated sex differences in Ep-CAM, in healthy subjects or in patients.

LPL is an enzyme widely expressed in the heart which catalyzes the hydrolysis of triglyceride-rich lipoproteins to fatty acids, as fuel for cardiomyocyte metabolism [46]. It plays an important role in atherogenesis and has been associated with coronary heart disease, Alzheimer’s disease, and chronic lymphocytic leukemia [25]. LPL deficiency leads to hypertriglyceridemia [47], whereas overexpression of LPL in a mice model resulted in insulin resistance and obesity [48, 49]. Influencing cardiac LPL in diabetic mouse models (by either overexpression or downregulation) resulted in impaired cardiac function [24].

Another enzyme involved in lipid metabolism is PLIN1 which is a surface protein of adipocyte lipid droplets that regulates storage and hydrolysis of adipose triglycerides. It has been associated with metabolic diseases such as diabetes, obesity, hepatic steatosis, certain cancers, and cardiovascular disease [33]. Downregulation of PLIN1 in mice led to excessive cardiac hypertrophy and failure [50]. A previous study demonstrated that PLIN1, as a marker of myocardial steatosis, was higher expressed in the right atria of patients with CAD compared to those without [34].

LHB (the beta subunit of luteinizing hormone) promotes spermatogenesis and ovulation by stimulating the testes and ovaries to synthesize steroids. A previous study measured LH in men and postmenopausal women with CAD but also in controls and demonstrated that LH levels were higher in females compared to males but that there was no difference between CAD and control subjects [26]. It is therefore possible that our finding of higher LHB levels in female compared to male HFpEF patients may be explained by higher LHB in females in general and that it may not be HFpEF-specific.

Proteins with higher expression in male compared to female HFpEF patients were NRP, ACE2, and MMP-3. NRP1 is a transmembrane receptor for class III semaphorins and for members of the vascular endothelial growth factor family [37]. It plays a role in neuronal and vascular development during embryogenesis, angiogenesis, and maintenance of vascular integrity [38]. In a murine model of cardiac pressure overload, animals that were heterozygous for neuropilin showed higher mortality rates [51]. Additionally, Tromp et al. demonstrated that NRP1 was associated with poor outcome in HFpEF but not in HFrEF patients [39].

ACE2 hydrolyses angiotensin I and angiotensin II generating angiotensin (1–9) and angiotensin (1–7), respectively. Both angiotensin (1–9) and angiotensin (1–7) are believed to possess direct protective effects against cardiac remodeling and it is hypothesized that the failing heart overexpresses ACE2 to protect itself against the deleterious effects of angiotensin II [52]. There is however conflicting data on sex differences in ACE2 expression. One study demonstrated similar ACE2 levels between healthy male and female subjects [45], while another study demonstrated that male controls had significantly higher ACE2 levels compared to females [44]. Although still significantly higher in male compared to female patients, the latter study demonstrated also that ACE2 was significantly higher in patients with type 1 diabetes and coronary heart disease compared to controls [44]. In line with this, a recent study in heart failure with reduced ejection fraction patients also showed that ACE2 was higher in male than female patients, independent of the use of pharmacological therapies targeting the renin–angiotensin–aldosterone system [53]. It is clear that further study to sex differences in ACE2 is warranted.

MMP-3 (or stromelysin-1) is an enzyme involved in the breakdown of extracellular matrix proteins and tissue remodeling in physiological (e.g., embryogenesis) and pathophysiological (e.g., tumor metastasis and atherosclerosis) processes [40]. High MMP-3 levels have been described in atherosclerotic plaques [41]. In contrast, a common mutation in the MMP-3 promoter (which results in decreased MMP-3 expression) was associated with atherosclerosis development [43]. Also, in patients with advanced dilated cardiomyopathy, MMP-3 levels were undetectable suggesting that lower levels are associated with worse cardiac function [54]. However, in a study of post-MI patients, MMP-3 levels were higher in male compared to female patients and higher MMP-3 levels were associated with poor outcome in MI [42]. In our study, we found that MMP-3 levels were higher in male than in female HFpEF patients. It is obvious that the exact role of MMP-3 remains unclear and warrants further investigation.

It must be noted that one of the circulating proteins that was excluded from the final selection because of being correlated to a different protein (PLIN1 in this case) was leptin. Leptin is a hormone that is released by adipocytes when they are overfilled with lipids, causing reduced food intake and increased energy expenditure [55]. It activates the sympathetic nervous system resulting in unfavorable neurohormonal changes such as an increase in blood pressure and cardiac hypertrophy [56]. A role in the pathophysiology of HFpEF was also suggested In HF patients (both HFrEF as HFpEF), and leptin levels were higher as compared to healthy controls [57]. Interestingly, the actions of leptin on the autonomic nervous system appear to be particularly marked in women [58]. In our study, female HFpEF patients had higher leptin levels compared to male patients. Although it has been previously hypothesized that leptin may play a role in the pathophysiology of HFpEF [59], the role of sex differences herein are not yet fully understood.

As can be appreciated above, for most of the circulating proteins that were differentially expressed between female and male HFpEF patients in our study, we could not find previous studies on sex differences. However, there have been reports on sex differences for some of the observed pathways. Body fat distribution is different between sexes where women have more subcutaneous and men more visceral fat. Visceral adiposity, as compared to subcutaneous adiposity, is associated with increased rates of lipolysis and inflammation, hence resulting in an increased susceptibility for metabolic complications (as reviewed in [60]). In HFpEF, it is hypothesized comorbidities such as diabetes and obesity induce a low-grade systemic pro-inflammatory state [61]. Whether sex differences in lipid metabolism are of importance in the pathophysiology of HFpEF warrants further investigation.

Moreover, there is an increasing body of evidence suggesting sex differences in primary and secondary hemostasis and fibrinolysis. It has been demonstrated, for example, that women have higher platelet counts and activity and generate more thrombin compared to men (as reviewed in [62]).

Lastly, sex differences in extracellular matrix turnover have also been previously reported. Women demonstrate a smaller fibrotic response compared to men as reflected by a lower expression of TGF-beta (a highly pro-fibrotic growth factor) in women (as reviewed in [63]). Moreover, in a mouse model, matrix metalloproteinase activity was higher in males compared to females after an acute MI, leading to poorer outcome in males [64]. Although cardiac fibrosis has been hypothesized by Paulus et al. to be one of the key players in the pathophysiology of HFpEF [61], the role of sex differences herein has yet to be identified. It is also possible that our finding of more activated extracellular matrix pathways in male compared to female HFpEF patients simply reflects the presence of more CAD in males (as observed in our study) and subsequent myocardial injury-induced extracellular matrix alterations.

### Sex differences: outcome

Female sex was not associated with cardiovascular death and/or cardiovascular hospitalizations in our study. Our findings are in line with a recent post hoc analysis from PARAGON demonstrating similar rates of the composite of heart failure hospitalizations or death for cardiovascular reasons [65], but in conflict with previous other studies. Lam et al. observed in a post hoc analysis of I-PRESERVE that female sex was associated with better prognosis, although the effect was moderated by 4 common baseline characteristics which were atrial fibrillation, renal dysfunction, stable angina pectoris, and NYHA class III/IV [20]. In the Meta-Analysis Global Group in Chronic Heart Failure (MAGGIC) meta-analysis, women had lower rates of all-cause mortality over 3 years compared to men, irrespective of ejection fraction [66]. Our findings and those of Lam et al. suggest that the relation between sex and outcome in HFpEF is confounded to some extent by comorbidities or other patient characteristics. Future studies need to determine whether a sex difference in prognosis exists.

### Perspectives and significance

For the majority of the circulating proteins that were differently expressed between female and male HFpEF patients in our study, sex differences in a healthy population have not been studied. A recent population-based study compared 30 cardiometabolic biomarkers (not including the proteins that were measured in MEDIA-DHF) between female and male participants and found that biomarker profiles significantly differed [67]. Whether the sex differences in circulating proteins in our cohort of HFpEF patients reflect pathophysiological or physiological processes warrants further study. Our findings of different pathways being activated in female compared to male HFpEF patients may be the basis of investigating therapies specifically targeting the identified pathways, potentially remediating the poor track record of past HFpEF large outcome trials [68]. In addition, they may be of help in the search for biologically plausible explanations for the sex differences in treatment response as reported for neprilysin inhibition [69], mineralocorticoid receptor antagonists [70], and cardiac resynchronization therapy [71].

### Limitations

The most important limitation of this study is that we could not externally validate our findings due to the absence of other HFpEF cohorts in which similar proteins were measured. It is clear that other studies need to confirm our findings. Second, as already mentioned before, sex differences have not been studied in a general population for the majority of circulating proteins tested in our study. Moreover, in this study, only HFpEF patients were included and a “healthy” control group (including reference values for the measured proteins) was thus absent. It is therefore not clear whether the observed differences in biomarker expression reflect pathophysiological and/or physiological processes. Third, at the time of enrollment, HFpEF was diagnosed following 2007 ESC diagnostic recommendations. Since the 2007 criteria, other criteria have been proposed and it has recently been shown that large variations in the prevalence of diastolic dysfunction may be expected according to which criterion is used [72]. However, when applying the H_2_FPEF score by Reddy and colleagues [73] to the MEDIA-DHF cohort, only 2% of patients had a low probability of HFpEF, whereas 58% and 40% of patients had an intermediate or high probability, respectively. Fourth, although the biomarker assay in this study covers a wide variety of disease domains, the possibility exists that other, now unmeasured, proteins may (also) play a role in the pathophysiology of HFpEF. Fifth, the biomarker assay does not provide standard concentration units, making comparisons with clinically applied cut-offs difficult.

## Conclusion

Assessing sex differences in > 400 circulating proteins analyzed in a large cohort of HFpEF patients led to the identification of 9 uncorrelated sex-specific proteins. Females expressed more markers associated with lipid metabolism, transcriptional regulation, and hemostasis, whereas they expressed less proteins associated with extracellular matrix organization and developmental processes. These findings may help further investigations into potential pathophysiological processes contributing to HFpEF.

## Supplementary information

**Additional file 1:** Supplemental data. Table S1-S3.

**Additional file 2.** Flowchart of patients included in the study

## Data Availability

The data that support the findings of this study are available from the corresponding author upon reasonable request.
