# Supplementary material for: Sex differences in circulating proteins in heart failure with preserved ejection fraction
Source: Biol Sex Differ. 2020 Aug 24;11:47. doi: 10.1186/s13293-020-00322-7 (PMC7444077; doi:10.1186/s13293-020-00322-7)
Supplement: Supplementary file 1 — Additional file 1: Supplemental data. Table S1-S3. [file 13293_2020_322_MOESM1_ESM.docx]

***Supplemental data***

***Echocardiography***

All patients underwent echocardiography and images were stored in a digital cine-loop format for off-line analysis according to the American Society of Echocardiography and the European Association of Cardiovascular Imaging relative to the cardiac chamber and right heart measurements^[[1]](#footnote-1)^

LV structure and function were evaluated from standard 2D views, including apical four-chamber, apical two-chamber and parasternal long- and short-axis views. Ventricular dimensions, wall thickness, mass and geometry were determined from 2D parasternal short- and long-axis views. LV volumes, stroke volume and ejection fraction were calculated using the biplane method of disks summation (modified Simpson’s rule). All cardiac chamber volumes and mass measurements were indexed to body surface area.

Left atrial volume was assessed by the biplane area-length method from apical 2- and 4-chamber views at end-systole and was indexed to body surface area (LA volume index, LAVi). Left atrial area (cm²) was estimated from the apical views. If LA volume was not available, LA area was used to evaluate remodeling. Patients with either LAVi>40ml/m² or LA area>20cm^2^ were considered to have dilated LA.

Peak velocity of early (E) and late (A) wave of transmitral flow and E-wave deceleration time (DT) were measured from the pulsed-wave Doppler obtained at the tip of mitral leaflets. The average of septal and lateral annular velocities (e’) was obtained by tissue Doppler imaging (TDI). The E/e′ ratio was calculated using the peak E-wave velocity and the average of septal and lateral. LV outflow tract (LVOT) time velocity integral, isovolumic relaxation time (IVRT) and color M-mode of early diastolic mitral inflow into the left ventricle (Flow propagation velocity, Vp) were acquired from apical four- and five-chamber views. Pulmonary venous flow (PVF) was sampled using pulsed-wave Doppler at 1 cm into the orifice of the right upper pulmonary vein.

Right ventricular function was estimated by measuring TAPSE (mm) and pulmonary arterial systolic pressure (PASP) was calculated using the peak velocity of tricuspid regurgitation (TR) and the maximum IVC diameter (IVC baseline) and respiratory variation (Ratio IVC inspiration / IVC baseline) measured 3 cm before merger with the right atrium.

Overall, echocardiographic data were complete in >75% of patients, except for the following variables: A (and E/A ratio), S/D, Ard-Ad, E/Vp (which were available in 70%, 61%, 44% and 62% of the population, respectively).

***Supplemental table 1 Participating centers MEDIA-DHF***

| Nancy Clinical Investigation Centre, University of Lorraine, France (and EDDH European Drug Development Hub, Nancy, France as project manager) |
| --- |
| Lariboisière Hospital, Paris, France |
| Free University Medical Centre, Amsterdam, The Netherlands |
| CHARITE, universitätsmedizin Berlin, Berlin Germany |
| University of Porto, Porto, Portugal |
| University of Maastricht, Maastricht, The Netherlands |
| University of Antwerp, Antwerp, Belgium |
| Cardiff University, Cardiff, United Kingdom |
| Foundation for Applied Medical Research, Pamplona, Spain |
| University of Eastern PIEMONTE Medical School, Novara, Italy |
| University of Brescia, Brescia, Italy |
| University of Perugia, Perugia, Italy |
| University College Dublin, National University of Ireland, Dublin, Ireland |
| University Debrecen, Debrecen, Hungary |
| Oslo University Hospital |

***Supplemental table 2 Overview of circulating proteins measured at baseline in MEDIA-DHF***

| **Organ damage panel** |  |
| --- | --- |
| 5'-AMP-activated protein kinase subunit beta-1 (PRKAB1) Q9Y478 | Kidney Injury Molecule (KIM1) Q96D42 |
| Adhesion G-protein coupled receptor G1 (ADGRG1) Q9Y653 | Leukotriene A-4 hydrolase (LTA4H) P09960 |
| Aldehyde dehydrogenase, dimeric NADP-preferring (ALDH3A1) P30838 | Linker for activation of T-cells family member 2 (LAT2) Q9GZY6 |
| Anterior gradient protein 2 homolog (AGR2) O95994 | Lutropin subunit beta (LHB) P01229 |
| Apoptosis-inducing factor 1, mitochondrial (AIFM1) O95831 | Macrophage erythroblast attacher (MAEA) Q7L5Y9 |
| B-cell scaffold protein with ankyrin repeats (BANK1) Q8NDB2 | Macrophage-capping protein (CAPG) P40121 |
| BH3-interacting domain death agonist (BID) P55957 | Melanoma-associated antigen D1 (MAGED1) Q9Y5V3 |
| BMP and activin membrane-bound inhibitor homolog (BAMBI) Q13145 | Methionine aminopeptidase 1 (METAP1) P53582 |
| Calcitonin (CALCA) P01258 | Mevalonate kinase (MVK) Q03426 |
| Calreticulin (CALR) P27797 | Mitogen-activated protein kinase kinase kinase kinase 5 (MAP4K5) Q9Y4K4 |
| Carbonic anhydrase 12 (CA12) O43570 | Mothers against decapentaplegic homolog 1 (SMAD1) Q15797 |
| Carbonic anhydrase 14 (CA14) Q9ULX7 | NAD-dependent protein deacylase sirtuin-5, mitochondrial (SIRT5) Q9NXA8 |
| Casein kinase I isoform delta (CSNK1D) P48730 | NEDD8 ultimate buster 1 (NUB1) Q9Y5A7 |
| Claspin (CLSPN) Q9HAW4 | Neutrophil cytosol factor 2 (NCF2) P19878 |
| CMP-N-acetylneuraminate-beta-galactosamide-alpha-2,3- | Nibrin (NBN) O60934 |
| sialyltransferase 1 (ST3GAL1) | Nitric oxide synthase, endothelial (NOS3) P29474 |
| Q11201 | Nucleobindin-2 (NUCB2) P80303 |
| Cocaine esterase (CES2) O00748 | Parvalbumin alpha (PVALB) P20472 |
| Contactin-2 (CNTN2) Q02246 | Paxillin (PXN) P49023 |
| Corticoliberin (CRH) P06850 | Peptidyl-prolyl cis-trans isomerase FKBP1B (FKBP1B) P68106 |
| C-type lectin domain family 1 member A (CLEC1A) Q8NC01 | Perilipin-1 (PLIN1) O60240 |
| C-type natriuretic peptide (NPPC) P23582 | Placenta growth factor (PGF) P49763 |
| Desmoglein-4 (DSG4) Q86SJ6 | Platelet-derived growth factor C (PDGFC) Q9NRA1 |
| Dipeptidyl aminopeptidase-like protein 6 (DPP6) P42658 | Pleiotrophin (PTN) P21246 |
| DNA topoisomerase 2-beta (TOP2B) Q02880 | Phosphatidylinositol 3,4,5-trisphosphate 5-phosphatase 2 (INPPL1) O15357 |
| Ectonucleoside triphosphate diphosphohydrolase 2 (ENTPD2) Q9Y5L3 | Plexin domain-containing protein 1 (PLXDC1) Q8IUK5 |
| Ectonucleoside triphosphate diphosphohydrolase 6 (ENTPD6) O75354 | Polypeptide N-acetylgalactosaminyltransferase 10 (GALNT10) Q86SR1 |
| EGF-like repeat and discoidin I-like domain-containing protein 3 (EDIL3) O43854 | Probetacellulin (BTC) P35070 |
| Enteropeptidase (TMPRSS15) P98073 | Programmed cell death protein 1 (PDCD1) Q15116 |
| Epidermal growth factor-like protein 7 (EGFL7) Q9UHF1 | Prolow-density lipoprotein receptor-related protein 1 (LRP1) Q07954 |
| Erbin (ERBIN) Q96RT1 | Proteasome subunit alpha type-1 (PSMA1) P25786 |
| Erythropoietin (EPO) P01588 | Protein amnionless (AMN) Q9BXJ7 |
| Fatty acid-binding protein 9 (FABP9) Q0Z7S8 | Protein fosB (FOSB) P53539 |
| Forkhead box protein O1 (FOXO1) Q12778 | Protein max (MAX) P61244 |
| Fructose-2,6-bisphosphatase TIGAR (TIGAR) Q9NQ88 | Protein phosphatase 1B (PPM1B) O75688 |
| Hematopoietic prostaglandin D synthase (HPGDS) O60760 | [Pyruvate dehydrogenase [acetyl-transferring]]-phosphatase 1, |
| Inactive tyrosine-protein kinase 7 (PTK7) Q13308 | mitochondrial (PDP1) Q9P0J1 |
| Integrin beta-1-binding protein 1 (ITGB1BP1) O14713 | Ras association domain-containing protein 2 (RASSF2) P50749 |
| Interferon-inducible double-stranded RNA-dependent protein | Ras GTPase-activating protein 1 (RASA1) P20936 |
| kinase activator A (PRKRA) O75569 | Receptor-type tyrosine-protein phosphatase eta (PTPRJ) Q12913 |
| Renin receptor (ATP6AP2) O75787 | EGF-containing fibulin-like extracellular matrix protein 1 (EFEMP1) Q12805 |
| REST corepressor 1 (RCOR1) Q9UKL0 | Endoglin (ENG) P17813 |
| Retinoic acid receptor responder protein 1 (RARRES1) P49788 | Fetuin-B (FETUB) Q9UGM5 |
| Ribonucleoside-diphosphate reductase subunit M2 B (RRM2B) Q7LG56 | Ficolin-2 (FCN2) Q15485 |
| Serpin A9 (SERPINA9) Q86WD7 | Glutaminyl-peptide cyclotransferase (QPCT) Q16769 |
| Serum paraoxonase/arylesterase 2 (PON2) Q15165 | Granulysin (GNLY) P22749 |
| Syntaxin-8 (STX8) Q9UNK0 | Growth arrest-specific protein 6 (GAS6) Q14393 |
| Syntaxin-binding protein 3 (STXBP3) O00186 | Hepatocyte growth factor receptor (MET) P08581 |
| Troponin I, cardiac muscle (TNNI3) P19429 | Ig lambda-2 chain C regions (IGLC2) P0CG05 |
| Tyrosine-protein kinase Fes/Fps (FES) P07332 | Insulin-like growth factor-binding protein 3 (IGFBP3) P17936 |
| Tyrosine-protein kinase Fgr (FGR) P09769 | Insulin-like growth factor-binding protein 6 (IGFBP6) P24592 |
| Tyrosine-protein kinase Yes (YES1) P07947 | Integrin alpha-M (ITGAM) P11215 |
| Vascular endothelial growth factor C (VEGFC) P49767 | Intercellular adhesion molecule 1 (ICAM1) P05362 |
| Vasohibin-1 (VASH1) Q7L8A9 | Intercellular adhesion molecule 3 (ICAM3) P32942 |
| Wiskott-Aldrich syndrome protein (WAS) P42768 | Interleukin-7 receptor subunit alpha (IL7R) P16871 |
|  | Latent-transforming growth factor beta-binding protein 2 (LTBP2) Q14767 |
| **Cardiometabolic panel** | Leukocyte immunoglobulin-like receptor subfamily B member 1 (LILRB1) Q8NHL6 |
| Angiogenin (ANG) P03950 | Leukocyte immunoglobulin-like receptor subfamily B member 2 (LILRB2) Q8N423 |
| Angiopoietin-related protein 3 (ANGPTL3) Q9Y5C1 | Leukocyte immunoglobulin-like receptor subfamily B member 5 (LILRB5) O75023 |
| Apolipoprotein M (APOM) O95445 | Lithostathine-1-alpha (REG1A) P05451 |
| Beta-Ala-His dipeptidase (CNDP1) Q96KN2 | Liver carboxylesterase 1 (CES1) P23141 |
| Beta-galactoside alpha-2,6-sialyltransferase 1 (ST6GAL1) P15907 | Low affinity immunoglobulin gamma Fc region receptor II-a (FCGR2A) P12318 |
| Cadherin-1 (CDH1) P12830 | Low affinity immunoglobulin gamma Fc region receptor III-B (FCGR3B) O75015 |
| Carbonic anhydrase 1 (CA1) P00915 | L-selectin (SELL) P14151 |
| Carbonic anhydrase 3 (CA3) P07451 | Lymphatic vessel endothelial hyaluronic acid receptor 1 (LYVE1) Q9Y5Y7 |
| Carbonic anhydrase 4 (CA4) P22748 | Lysosomal Pro-X carboxypeptidase (PRCP) P42785 |
| Cartilage acidic protein 1 (CRTAC1) Q9NQ79 | Mannose-binding protein C (MBL2) P11226 |
| Cartilage oligomeric matrix protein (COMP) P49747 | Mast/stem cell growth factor receptor Kit (KIT) P10721 |
| C-C motif chemokine 5 (CCL5) P13501 | Membrane cofactor protein (CD46) P15529 |
| C-C motif chemokine 14 (CCL14) Q16627 | Membrane primary amine oxidase (AOC3) Q16853 |
| C-C motif chemokine 18 (CCL18) P55774 | Metalloproteinase inhibitor 1 (TIMP1) P01033 |
| CD59 glycoprotein (CD59) P13987 | Microfibrillar-associated protein 5 (MFAP5) Q13361 |
| Coagulation factor VII (F7) P08709 | Multiple epidermal growth factor-like domains protein 9 (MEGF9) Q9H1U4 |
| Coagulation factor XI (F11) P03951 | Neural cell adhesion molecule 1 (NCAM1) P13591 |
| Collagen alpha-1(XVIII) chain (COL18A1) P39060 | Neural cell adhesion molecule L1-like protein (CHL1) O00533 |
| Complement C1q tumor necrosis factor-related protein 1 (C1QTNF1) Q9BXJ1 | Neurogenic locus notch homolog protein 1 (NOTCH1) P46531 |
| Complement C2 (C2) P06681 | Neuropilin-1 (NRP1) O14786 |
| Complement factor H-related protein 5 (CFHR5) Q9BXR6 | Neutrophil defensin 1 (DEFA1) P59665 |
| Complement receptor type 2 (CR2) P20023 | Neutrophil gelatinase-associated lipocalin (LCN2) P80188 |
| Cystatin-C (CST3) P01034 | Nidogen-1 (NID1) P14543 |
| Dipeptidyl peptidase 4 (DPP4) P27487 | Oncostatin-M-specific receptor subunit beta (OSMR) Q99650 |
| Peptidyl-glycine alpha-amidating monooxygenase (PAM) P19021 | C-C motif chemokine 17 (CCL17) Q92583 |
| Phospholipid transfer protein (PLTP) P55058 | CD40 ligand (CD40-L) P29965 |
| Plasma serine protease inhibitor (SERPINA5) P05154 | Chymotrypsin C (CTRC) Q99895 |
| Platelet glycoprotein Ib alpha chain (GP1BA) P07359 | C-X-C motif chemokine 1 (CXCL1) P09341 |
| Platelet-activating factor acetylhydrolase (PLA2G7) Q13093 | Decorin (DCN) P07585 |
| Plexin-B2 (PLXNB2) O15031 | Dickkopf-related protein 1 (Dkk-1) O94907 |
| Procollagen C-endopeptidase enhancer 1 (PCOLCE) Q15113 | Fatty acid-binding protein, intestinal (FABP2) P12104 |
| Prolyl endopeptidase FAP (FAP) Q12884 | Fibroblast growth factor 21 (FGF-21) Q9NSA1 |
| Receptor-type tyrosine-protein phosphatase S (PTPRS) Q13332 | Fibroblast growth factor 23 (FGF-23) Q9GZV9 |
| Regenerating islet-derived protein 3-alpha (REG3A) Q06141 | Follistatin (FS) P19883 |
| Serum amyloid A-4 protein (SAA4) P35542 | Galectin-9 (Gal-9) O00182 |
| SPARC-like protein 1 (SPARCL1) Q14515 | Gastric intrinsic factor (GIF) P27352 |
| Superoxide dismutase [Cu-Zn] (SOD1) P00441 | Gastrotropin (GT) P51161 |
| T-cell immunoglobulin and mucin domain-containing protein 4 (TIMD4) Q96H15 | Growth hormone (GH) P01241 |
| Tenascin (TNC) P24821 | Growth/differentiation factor 2 (GDF-2) Q9UK05 |
| Tenascin-X (TNXB) P22105 | Heat shock 27 kDa protein (HSP 27) P04792 |
| Thrombospondin-4 (THBS4) P35443 | Heme oxygenase 1 (HO-1) P09601 |
| Thyroxine-binding globulin (SERPINA7) P05543 | Hydroxyacid oxidase 1 (HAOX1) Q9UJM8 |
| Transcobalamin-2 (TCN2) P20062 | Interleukin-1 receptor antagonist protein (IL-1ra) P18510 |
| Transforming growth factor beta receptor type 3 (TGFBR3) Q03167 | Interleukin-1 receptor-like 2 (IL1RL2) Q9HB29 |
| Transforming growth factor-beta-induced protein ig-h3 (TGFBI) Q15582 | Interleukin-4 receptor subunit alpha (IL-4RA) P24394 |
| Trypsin-2 (PRSS2) P07478 | Interleukin-6 (IL6) P05231 |
| Tyrosine-protein kinase receptor Tie-1 (TIE1) P35590 | Interleukin-17D (IL-17D) Q8TAD2 |
| Uromodulin (UMOD) P07911 | Interleukin-18 (IL-18) Q14116 |
| Vascular cell adhesion protein 1 (VCAM1) P19320 | Interleukin-27 (IL-27) Q8NEV9, |
| Vasorin (VASN) Q6EMK4 | Q14213 |
| Vitamin K-dependent protein C (PROC) P04070 | Kidney Injury Molecule (KIM1) Q96D42 |
|  | Lactoylglutathione lyase (GLO1) Q04760 |
| **Cardiovascular II panel** | Lectin-like oxidized LDL receptor 1 (LOX-1) P78380 |
| 2,4-dienoyl-CoA reductase, mitochondrial (DECR1) Q16698 | Leptin (LEP) P41159 |
| A disintegrin and metalloproteinase with thrombospondin motifs 13 (ADAM-TS13) Q76LX8 | Lipoprotein lipase (LPL) P06858 |
| ADM (ADM) P35318 | Low affinity immunoglobulin gamma Fc region receptor II-b (IgG Fc receptor II-b) P31994 |
| Agouti-related protein (AGRP) O00253 | Lymphotactin (XCL1) P47992 |
| Alpha-L-iduronidase (IDUA) P35475 | Macrophage receptor MARCO (MARCO) Q9UEW3 |
| Angiopoietin-1 (ANG-1) Q15389 | Matrix metalloproteinase-7 (MMP-7) P09237 |
| Angiopoietin-1 receptor (TIE2) Q02763 | Matrix metalloproteinase-12 (MMP-12) P39900 |
| Angiotensin-converting enzyme 2 (ACE2) Q9BYF1 | Melusin (ITGB1BP2) Q9UKP3 |
| Bone morphogenetic protein 6 (BMP-6) P22004 | Natriuretic peptides B (BNP) P16860 |
| Brother of CDO (Protein BOC) Q9BWV1 | NF-kappa-B essential modulator (NEMO) Q9Y6K9 |
| Carbonic anhydrase 5A, mitochondrial (CA5A) P35218 | Osteoclast-associated immunoglobulin-like receptor (hOSCAR) Q8IYS5 |
| Carcinoembryonic antigenrelated cell adhesion molecule 8 (CEACAM8) P31997 | Pappalysin-1 (PAPPA) Q13219 |
| Cathepsin L1 (CTSL1) P07711 | Pentraxin-related protein PTX3 (PTX3) P26022 |
| C-C motif chemokine 3 (CCL3) P10147 | Placenta growth factor (PGF) P49763 |
| Platelet-derived growth factor subunit B (PDGF subunit B) P01127 | Caspase-3 (CASP-3) P42574 |
| Poly [ADP-ribose] polymerase 1 (PARP-1) P09874 | Cathepsin D (CTSD) P07339 |
| Polymeric immunoglobulin receptor (PIgR) P01833 | Cathepsin Z (CTSZ) Q9UBR2 |
| Programmed cell death 1 ligand 2 (PD-L2) Q9BQ51 | C-C motif chemokine 15 (CCL15) Q16663 |
| Proheparin-binding EGF-like growth factor (HB-EGF) Q99075 | C-C motif chemokine 16 (CCL16) O15467 |
| Pro-interleukin-16 (IL16) Q14005 | Note: New assay under development N/A |
| Prolargin (PRELP) P51888 | C-C motif chemokine 24 (CCL24) O00175 |
| Prostasin (PRSS8 ) Q16651 | CD166 antigen (ALCAM) Q13740 |
| Protein AMBP (AMBP) P02760 | Chitinase-3-like protein 1 (CHI3L1) P36222 |
| Proteinase-activated receptor 1 (PAR-1) P25116 | Chitotriosidase-1 (CHIT1) Q13231 |
| Protein-glutamine gamma-glutamyltransferase 2 (TGM2) P21980 | Collagen alpha-1(I) chain (COL1A1) P02452 |
| Proto-oncogene tyrosine-protein kinase Src (SRC) P12931 | Complement component C1q receptor (CD93) Q9NPY3 |
| P-selectin glycoprotein ligand 1 (PSGL-1) Q14242 | Contactin-1 (CNTN1) Q12860 |
| Receptor for advanced glycosylation end products (RAGE) Q15109 | C-X-C motif chemokine 16 (CXCL16) Q9H2A7 |
| Renin (REN) P00797 | Cystatin-B (CSTB) P04080 |
| Serine protease 27 (PRSS27) Q9BQR3 | Elafin (PI3) P19957 |
| Serine/threonine-protein kinase 4 (STK4) Q13043 | Ephrin type-B receptor 4 (EPHB4) P54760 |
| Serpin A12 (SERPINA12) Q8IW75 | Epidermal growth factor receptor (EGFR) P00533 |
| SLAM family member 5 (CD84) Q9UIB8 | Epithelial cell adhesion molecule (Ep-CAM) P16422 |
| SLAM family member 7 (SLAMF7) Q9NQ25 | E-selectin (SELE) P16581 |
| Sortilin (SORT1) Q99523 | Fatty acid-binding protein, adipocyte (FABP4) P15090 |
| Spondin-2 (SPON2) Q9BUD6 | Galectin-3 (Gal-3) P17931 |
| Stem cell factor (SCF) P21583 | Galectin-4 (Gal-4) P56470 |
| Superoxide dismutase [Mn], mitochondrial (SOD2) P04179 | Granulins (GRN) P28799 |
| T-cell surface glycoprotein CD4 (CD4) P01730 | Growth/differentiation factor 15 (GDF-15) Q99988 |
| Thrombomodulin TM P07204 | Insulin-like growth factor-binding protein 1 (IGFBP-1) P08833 |
| Thrombopoietin (THPO) P40225 | Insulin-like growth factor-binding protein 2 (IGFBP-2) P18065 |
| Thrombospondin-2 (THBS2) P35442 | Insulin-like growth factor-binding protein 7 (IGFBP-7) Q16270 |
| Tissue factor (TF) P13726 | Integrin beta-2 (ITGB2) P05107 |
| TNF-related apoptosis-inducing ligand receptor 2 (TRAIL-R2) O14763 | Intercellular adhesion molecule 2 (ICAM-2) P13598 |
| Tumor necrosis factor receptor superfamily member 10A (TNFRSF10A) O00220 | Interleukin-1 receptor type 1 (IL-1RT1) P14778 |
| Tumor necrosis factor receptor superfamily member 11A (TNFRSF11A) Q9Y6Q6 | Interleukin-1 receptor type 2 (IL-1RT2) P27930 |
| Tumor necrosis factor receptor superfamily member 13B (TNFRSF13B) O14836 | Interleukin-2 receptor subunit alpha (IL2-RA) P01589 |
| Tyrosine-protein kinase Mer (MERTK) Q12866 | Interleukin-6 receptor subunit alpha (IL-6RA) P08887 |
| Vascular endothelial growth factor D (VEGFD) O43915 | Interleukin-17 receptor A (IL-17RA) Q96F46 |
| V-set and immunoglobulin domain-containing protein 2 (VSIG2) Q96IQ7 | Interleukin-18-binding protein (IL-18BP) O95998 |
| **Cardiovascular III panel** | Junctional adhesion molecule A (JAM-A) Q9Y624 |
| Aminopeptidase N (AP-N) P15144 | Kallikrein-6 (KLK6) Q92876 |
| Azurocidin (AZU1 P20160 | Low-density lipoprotein receptor (LDL receptor) P01130 |
| Bleomycin hydrolase (BLM hydrolase) Q13867 | Lymphotoxin-beta receptor (LTBR) P36941 |
| Cadherin-5 (CDH5) P33151 | Matrix extracellular phosphoglycoprotein (MEPE) Q9NQ76 |
| Carboxypeptidase A1 (CPA1) P15085 | Matrix metalloproteinase-2 (MMP-2) P08253 |
| Carboxypeptidase B (CPB1) P15086 | Matrix metalloproteinase-3 (MMP-3) P08254 |
| Matrix metalloproteinase-9 (MMP-9) P14780 | von Willebrand factor (vWF) P04275 |
| Metalloproteinase inhibitor 4 (TIMP4) Q99727 |  |
| Monocyte chemotactic protein 1 (MCP-1) P13500 | **Inflammation panel** |
| Myeloblastin (PRTN3) P24158 | Adenosine Deaminase (ADA) P00813 |
| Myeloperoxidase (MPO) P05164 | Artemin (ARTN) Q5T4W7 |
| Myoglobin (MB) P02144 | Axin-1 (AXIN1) O15169 |
| Neurogenic locus notch homolog protein 3 (Notch 3) Q9UM47 | Beta-nerve growth factor (Beta-NGF) P01138 |
| N-terminal prohormone brain natriuretic peptide (NT-proBNP) NA | Caspase-8 (CASP-8) Q14790 |
| Osteopontin (OPN) P10451 | C-C motif chemokine 3 (CCL3) P10147 |
| Osteoprotegerin (OPG) O00300 | C-C motif chemokine 4 (CCL4) P13236 |
| Paraoxonase (PON3) Q15166 | C-C motif chemokine 19 (CCL19) Q99731 |
| Peptidoglycan recognition protein 1 (PGLYRP1) O75594 | C-C motif chemokine 20 (CCL20) P78556 |
| Perlecan (PLC) P98160 | C-C motif chemokine 23 (CCL23) P55773 |
| Plasminogen activator inhibitor 1 (PAI) P05121 | C-C motif chemokine 25 (CCL25) O15444 |
| Platelet endothelial cell adhesion molecule (PECAM-1) P16284 | C-C motif chemokine 28 (CCL28) Q9NRJ3 |
| Platelet-derived growth factor subunit A (PDGF subunit A) P04085 | CD40L receptor (CD40) P25942 |
| Proprotein convertase subtilisin/kexin type 9 (PCSK9) Q8NBP7 | CUB domain-containing protein 1 (CDCP1) Q9H5V8 |
| Protein delta homolog 1 (DLK-1) P80370 | C-X-C motif chemokine 1 (CXCL1) P09341 |
| P-selectin (SELP) P16109 | C-X-C motif chemokine 5 (CXCL5) P42830 |
| Pulmonary surfactant-associated protein D (PSP-D) P35247 | C-X-C motif chemokine 6 (CXCL6) P80162 |
| Resistin (RETN) Q9HD89 | C-X-C motif chemokine 9 (CXCL9) Q07325 |
| Retinoic acid receptor responder protein 2 (RARRES2) Q99969 | C-X-C motif chemokine 10 (CXCL10) P02778 |
| Scavenger receptor cysteine-rich type 1 protein M130 (CD163) Q86VB7 | C-X-C motif chemokine 11 (CXCL11) O14625 |
| Secretoglobin family 3A member 2 (SCGB3A2) Q96PL1 | Cystatin D (CST5) P28325 |
| Spondin-1 (SPON1) Q9HCB6 | Delta and Notch-like epidermal growth factor-related receptor (DNER) Q8NFT8 |
| ST2 protein (ST2) Q01638 | Eotaxin (CCL11) P51671 |
| Tartrate-resistant acid phosphatase type 5 (TR-AP) P13686 | Eukaryotic translation initiation factor 4E-binding protein 1 (4E-BP1) Q13541 |
| Tissue factor pathway inhibitor (TFPI) P10646 | Fibroblast growth factor 21 (FGF-21) Q9NSA1 |
| Tissue-type plasminogen activator (t-PA) P00750 | Fibroblast growth factor 23 (FGF-23) Q9GZV9 |
| Transferrin receptor protein 1 (TR) P02786 | Fibroblast growth factor 5 (FGF-5) Q8NF90 |
| Trefoil factor 3 (TFF3) Q07654 | Fibroblast growth factor 19 (FGF-19) O95750 |
| Trem-like transcript 2 protein (TLT-2) Q5T2D2 | Fms-related tyrosine kinase 3 ligand (Flt3L) P49771 |
| Tumor necrosis factor ligand superfamily member 13B (TNFSF13B) Q9Y275 | Fractalkine (CX3CL1) P78423 |
| Tumor necrosis factor receptor 1 (TNF-R1) P19438 | Glial cell line-derived neurotrophic factor (GDNF) P39905 |
| Tumor necrosis factor receptor 2 (TNF-R2) P20333 | Hepatocyte growth factor (HGF) P14210 |
| Tumor necrosis factor receptor superfamily member 6 (FAS) P25445 | Interferon gamma (IFN-gamma) P01579 |
| Tumor necrosis factor receptor superfamily member 10C (TNFRSF10C) O14798 | Interleukin-1 alpha (IL-1 alpha) P01583 |
| Tumor necrosis factor receptor superfamily member 14 (TNFRSF14) Q92956 | Interleukin-2 (IL-2) P60568 |
| Tyrosine-protein kinase receptor UFO (AXL) P30530 | Interleukin-2 receptor subunit beta (IL-2RB) P14784 |
| Tyrosine-protein phosphatase non-receptor type substrate 1 (SHPS-1) P78324 | Interleukin-4 (IL-4) P05112 |
| Urokinase plasminogen activator surface receptor (U-PAR) Q03405 | Interleukin-5 (IL5) P05113 |
| Urokinase-type plasminogen activator (uPA) P00749 | Interleukin-6 (IL6) P05231 |
| Interleukin-7 (IL-7) P13232 | Thymic stromal lymphopoietin (TSLP) Q969D9 |
| Interleukin-8 (IL-8) P10145 | TNF-related activation-induced cytokine (TRANCE) O14788 |
| Interleukin-10 (IL10) P22301 | TNF-related apoptosis-inducing ligand (TRAIL) P50591 |
| Interleukin-10 receptor subunit alpha (IL-10RA) Q13651 | Transforming growth factor alpha (TGF-alpha) P01135 |
| Interleukin-10 receptor subunit beta (IL-10RB) Q08334 | Tumor necrosis factor (Ligand) superfamily, member 12(TWEAK) O43508 |
| Interleukin-12 subunit beta (IL-12B) P29460 | Tumor necrosis factor (TNF) P01375 |
| Interleukin-13 (IL-13) P35225 | Tumor necrosis factor ligand superfamily member 14 (TNFSF14) O43557 |
| Interleukin-15 receptor subunit alpha (IL-15RA) Q13261 | Tumor necrosis factor receptor superfamily member 9(TNFRSF9) Q07011 |
| Interleukin-17A (IL-17A) Q16552 | Urokinase-type plasminogen activator (uPA) P00749 |
| Interleukin-17C (IL-17C) Q9P0M4 | Vascular endothelial growth factor A (VEGF-A) P15692 |
| Interleukin-18 (IL-18) Q14116 |  |
| Interleukin-18 receptor 1 (IL-18R1) Q13478 |  |
| Interleukin-20 (IL-20) Q9NYY1 |  |
| Interleukin-20 receptor subunit alpha (IL-20RA) Q9UHF4 |  |
| Interleukin-22 receptor subunit alpha-1 (IL-22 RA1) Q8N6P7 |  |
| Interleukin-24 (IL-24) Q13007 |  |
| Interleukin-33 (IL-33) O95760 |  |
| Latency-associated peptide transforming growth factor beta-1 (LAP TGF-beta-1) P01137 |  |
| Leukemia inhibitory factor (LIF) P15018 |  |
| Leukemia inhibitory factor receptor (LIF-R) P42702 |  |
| Macrophage colony-stimulating factor 1 (CSF-1) P09603 |  |
| Matrix metalloproteinase-1 (MMP-1) P03956 |  |
| Matrix metalloproteinase-10 (MMP-10) P09238 |  |
| Monocyte chemotactic protein 1 (MCP-1) P13500 |  |
| Monocyte chemotactic protein 2 (MCP-2) P80075 |  |
| Monocyte chemotactic protein 3 (MCP-3) P80098 |  |
| Monocyte chemotactic protein 4 (MCP-4) Q99616 |  |
| Natural killer cell receptor 2B4 (CD244) Q9BZW8 |  |
| Neurotrophin-3 (NT-3) P20783 |  |
| Neurturin (NRTN) Q99748 |  |
| Oncostatin-M (OSM) P13725 |  |
| Osteoprotegerin (OPG) O00300 |  |
| Programmed cell death 1 ligand 1 (PD-L1) Q9NZQ7 |  |
| Protein S100-A12 (EN-RAGE) P80511 |  |
| Signaling lymphocytic activation molecule (SLAMF1) Q13291 |  |
| SIR2-like protein 2 (SIRT2) Q8IXJ6 |  |
| STAM-binding protein (STAMBP) O95630 |  |
| Stem cell factor (SCF) P21583 |  |
| Sulfotransferase 1A1 (ST1A1) P50225 |  |
| T cell surface glycoprotein CD6 isoform (CD6) Q8WWJ7 |  |
| T-cell surface glycoprotein CD5 (CD5) P06127 |  |
| T-cell surface glycoprotein CD8 alpha chain (CD8A) P01732 |  |
| TNF-beta (TNFB) P01374 |  |

***Supplemental table 2 Circulating protein concentrations (in NPX) according to sex***

|  | Global (N=392) | Male  (N=142) | Female  (N=250) | p-value |
| --- | --- | --- | --- | --- |
| IGFBP3 | 4.4 ± 0.6 | 4.2 ± 0.6 | 4.6 ± 0.5 | <0.001 |
| IL1RL2 | 3.2 ± 0.4 | 3.1 ± 0.4 | 3.3 ± 0.4 | <0.001 |
| LPL | 9.8 ± 0.6 | 9.5 ± 0.6 | 9.9 ± 0.5 | <0.001 |
| Ep-CAM | 4.8 ± 1.0 | 4.5 ± 0.8 | 5.0 ± 1.0 | <0.001 |
| PLIN1 | 2.2 ± 0.8 | 1.9 ± 0.6 | 2.3 ± 0.8 | <0.001 |
| LHB | 1.6 ± 1.1 | 1.1 ± 1.1 | 2.0 ± 1.0 | <0.001 |
| NRP1 | 2.2 ± 0.2 | 2.2 ± 0.2 | 2.1 ± 0.2 | <0.001 |
| MMP_3 | 5.9 ± 0.8 | 6.3 ± 0.7 | 5.6 ± 0.7 | <0.001 |
| ACE2 | 3.8 ± 0.7 | 4.1 ± 0.8 | 3.7 ± 0.6 | <0.001 |

Legend: IGFBP3, insulin-like growth factor-binding protein 3; NRP1, neuropilin 1; IL1RL2, interleukin-1 receptor-like 2; LPL, lipoprotein lipase; ACE2, angiotensin-converting-enzyme 2; Ep_CAM, epithelial cell adhesion molecule; CA14, carbonic anhydrase 14; PLIN1, perilipin-1; LHB, lutropin subunit beta.

***Supplemental Table 3 Circulating protein concentrations (in NPX) according to sex (after exclusion of proteins with correlation > 0.5)***

|  | Global (N=392) | Male  (N=142) | Female  (N=250) | p-value |
| --- | --- | --- | --- | --- |
| IGFBP3 | 4.4 ± 0.6 | 4.2 ± 0.6 | 4.6 ± 0.5 | <0.0001 |
| NRP1 | 2.2 ± 0.2 | 2.2 ± 0.2 | 2.1 ± 0.2 | <0.0001 |
| IL1RL2 | 3.2 ± 0.4 | 3.1 ± 0.4 | 3.3 ± 0.4 | <0.0001 |
| LPL | 9.8 ± 0.6 | 9.5 ± 0.6 | 9.9 ± 0.5 | <0.0001 |
| ACE2 | 3.8 ± 0.7 | 4.1 ± 0.8 | 3.7 ± 0.6 | <0.0001 |
| LEP | 5.8 ± 1.1 | 5.1 ± 1.1 | 6.2 ± 0.9 | <0.0001 |
| Gal_3 | 5.2 ± 0.4 | 5.0 ± 0.4 | 5.2 ± 0.4 | <0.0001 |
| Ep_CAM | 4.8 ± 1.0 | 4.5 ± 0.8 | 5.0 ± 1.0 | <0.0001 |
| MB | 7.2 ± 0.7 | 7.4 ± 0.7 | 7.0 ± 0.7 | <0.0001 |
| MMP_3 | 5.9 ± 0.8 | 6.3 ± 0.7 | 5.6 ± 0.7 | <0.0001 |
| CA14 | 1.2 ± 0.4 | 1.3 ± 0.4 | 1.1 ± 0.4 | <0.0001 |
| PLIN1 | 2.2 ± 0.8 | 1.9 ± 0.6 | 2.3 ± 0.8 | <0.0001 |
| CALCA | 4.7 ± 1.0 | 5.3 ± 1.0 | 4.4 ± 0.9 | <0.0001 |
| LHB | 1.6 ± 1.1 | 1.1 ± 1.1 | 2.0 ± 1.0 | <0.0001 |

Legend: IGFBP3, insulin-like growth factor-binding protein 3; NRP1, neuropilin 1; IL1RL2, interleukin-1 receptor-like 2; LPL, lipoprotein lipase; ACE2, angiotensin-converting-enzyme 2; LEP, leptin; Gal_3, galectin 3; Ep_CAM, epithelial cell adhesion molecule; MB, myoglobin, MMP_3, matrix metalloproteinase 3; CA14, carbonic anhydrase 14; PLIN1, perilipin 1; CALCA, calcitonin; LHB, lutropin subunit beta.

1. Lang RM *et al.* Recommendations for cardiac chamber quantification by echocardiography in adults: an update from the American Society of Echocardiography and the European Association of Cardiovascular Imaging. Eur Heart J Cardiovasc Imaging 2015; 16(3):233-70. [↑](#footnote-ref-1)
