# Supplementary figures and images for: Sex differences in circulating proteins in heart failure with preserved ejection fraction
Source: Biol Sex Differ. 2020 Aug 24;11:47. doi: 10.1186/s13293-020-00322-7 (PMC7444077; doi:10.1186/s13293-020-00322-7)

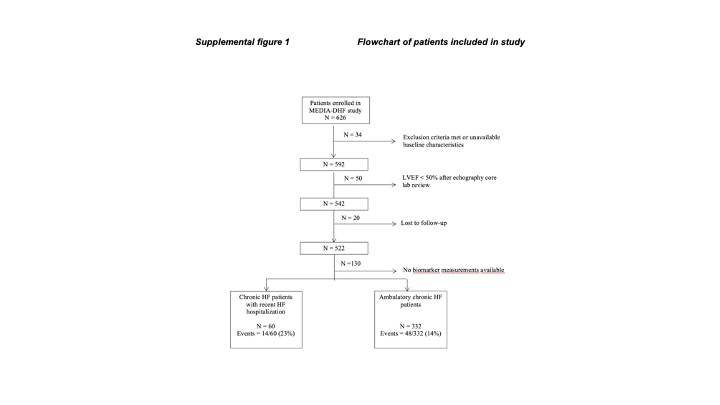

Supplement: Supplementary file 2 — Additional file 2. Flowchart of patients included in the study [file 13293_2020_322_MOESM2_ESM.jpg]
